# Phosphorylation by Cdk1 Increases the Binding of Eg5 to Microtubules *In Vitro* and in *Xenopus* Egg Extract Spindles

**DOI:** 10.1371/journal.pone.0003936

**Published:** 2008-12-15

**Authors:** Julie Cahu, Aurelien Olichon, Christian Hentrich, Henry Schek, Jovana Drinjakovic, Cunjie Zhang, Amanda Doherty-Kirby, Gilles Lajoie, Thomas Surrey

**Affiliations:** 1 European Molecular Biology Laboratory, Cell Biology and Biophysics Unit, Heidelberg, Germany; 2 Don Rix Protein Identification Facility, Schulich School of Medicine, The University of Western Ontario, London, Ontario, Canada; Duke University Medical Centre, United States of America

## Abstract

**Background:**

Motor proteins from the kinesin-5 subfamily play an essential role in spindle assembly during cell division of most organisms. These motors crosslink and slide microtubules in the spindle. Kinesin-5 motors are phosphorylated at a conserved site by Cyclin-dependent kinase 1 (Cdk1) during mitosis. *Xenopus laevis* kinesin-5 has also been reported to be phosphorylated by Aurora A *in vitro*.

**Methodology/Principal Findings:**

We investigate here the effect of these phosphorylations on kinesin-5 from *Xenopus laevis*, called Eg5. We find that phosphorylation at threonine 937 in the C-terminal tail of Eg5 by Cdk1 does not affect the velocity of Eg5, but strongly increases its binding to microtubules assembled in buffer. Likewise, this phosphorylation promotes binding of Eg5 to microtubules in *Xenopus* egg extract spindles. This enhancement of binding elevates the amount of Eg5 in spindles above a critical level required for bipolar spindle formation. We find furthermore that phosphorylation of *Xenopus laevis* Eg5 by Aurora A at serine 543 in the stalk is not required for spindle formation.

**Conclusions/Significance:**

These results show that phosphorylation of Eg5 by Cdk1 has a direct effect on the interaction of this motor with microtubules. In egg extract, phosphorylation of Eg5 by Cdk1 ensures that the amount of Eg5 in the spindle is above a level that is required for spindle formation. This enhanced targeting to the spindle appears therefore to be, at least in part, a direct consequence of the enhanced binding of Eg5 to microtubules upon phosphorylation by Cdk1. These findings advance our understanding of the regulation of this essential mitotic motor protein.

## Introduction

During cell division the duplicated chromosomes need to be segregated accurately to the new daughter cells, which is achieved in eukaryotic cells by a multi-component machine called the mitotic spindle. Bipolar spindle formation is initiated at the onset of mitosis due, in part, to an increase of kinase activities. In particular, the major cell cycle regulator Cyclin-dependent kinase 1 (Cdk1), previously also called *p34^cdc2^*, phosphorylates various proteins resulting in the reorganization of the intracellular architecture [1]. Other kinases such as Aurora or Polo and the small G protein Ran have also important regulatory roles during cell division [2], [3].

Microtubule associated proteins and molecular motors, cytoplasmic dynein and kinesins from various subfamilies, are essential for spindle formation and, hence, for chromosome segregation [4]–[6]. There is increasing evidence that molecular motors are temporally and spatially regulated, for example by phosphorylations [7], [8], interactions with Ran-dependent proteins [9] or degradation [10] to ensure their specific tasks at the correct time and at the correct location in the spindle.

Kinesin-5 is one of the most important motors for spindle assembly and integrity in almost all eukaryotes studied [11]–[15]. The loss of activity of Eg5, the kinesin-5 of vertebrates, prevents bipolar spindle assembly and causes the formation of monopolar spindles. In contrast to other kinesins, Eg5 is a homotetramer having two motor heads at each of the two ends of the elongated molecule [16]. Due to this bipolar structure, Eg5 can not only crosslink microtubules [16], [17], but also slide antiparallel microtubules *in vitro* [18], a property that is thought to be responsible for driving microtubule flux [19].

All members of the kinesin-5 subfamily have a conserved Cdk1 phosphorylation site at their C-terminus in the so called ‘bimC box’ [14]. Using immunofluorescence with phospho-specific antibodies, this site was reported to be phosphorylated on kinesin-5 bound to spindles in *Drosophila melanogaster* embryos [17]. Cell cycle dependent phosphorylation at this site has then been demonstrated to be essential for the mitotic function of kinesin-5 in *Drosophila melanogaster* S2 cells [20], but not in *Schizosaccharomyces pombe* [21]. Similar to the situation in *Drosophila*, non-phosphorylatable mutants of Eg5 do not localize to spindle microtubules when overexpressed in cultured human and *Xenopus* cells [7], [15]. The molecular mechanism by which Eg5 is targeted to spindle microtubules upon phosphorylation by Cdk1 is, however, unknown.

Eg5 was also shown to be phosphorylated *in vitro* by Aurora A at an unknown site in its stalk region [22]. Moreover, the kinase activity of Aurora A was reported to be essential for bipolar spindle formation, because addition of a catalytically inactive kinase leads to the formation of monopolar spindles in *Xenopus* egg extract, similar to the phenotype induced by inactivation or removal of Eg5 [23]. On the other hand, Eg5 was proposed to also be locally regulated by Ran.GTP in *Xenopus* egg extract [24]. This created the possibility that this proposed regulation by Ran.GTP could be mediated through the phosphorylation of Eg5 by Aurora A [25]–[27]. This kinase is activated [26], [28] and targeted to the spindle [29] by TPX2, which is released from importins by Ran.GTP [25]. The importance of the phosphorylation of Eg5 in its stalk region by Aurora A for spindle assembly has, however, not yet been tested directly.

In this study, we investigated the effect of phosphorylation on the activity of Eg5. We tested, if phosphorylation by Cdk1 modulates basic biophysical properties of Eg5 such as its efficiency of binding to microtubules and its velocity as measured *in vitro*. We found that Cdk1 strongly enhances the binding of purified Eg5 to microtubules. Using *Xenopus* egg extract, we showed that the cell cycle dependent regulation by Cdk1 is responsible for elevating the amount of Eg5 in the spindle above a critical value that needs to be exceeded to ensure spindle formation. We also identified a previously reported phosphorylation site in the stalk region of Eg5 by Aurora A [22] and found that it is not required for spindle formation in *Xenopus* egg extract, leaving the Cdk1 phosphorylation site in the tail of Eg5 as the only currently identified site whose phosphorylation is required for the mitotic function of Eg5.

## Results

**Figure 1:**
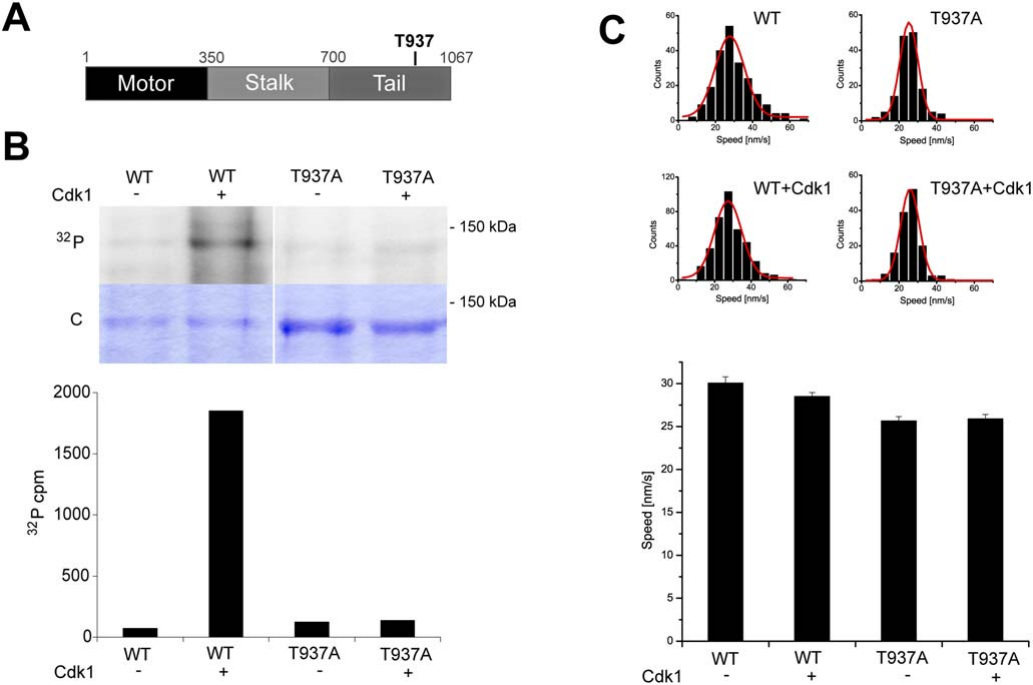
Phosphorylation of *Xenopus laevis* Eg5 by Cdk1/cyclin B *in vitro* does not affect its velocity as measured in microtubule gliding assays. (A) Schematic representation of the Eg5 sequence with the phosphorylation site for Cdk1 in the tail (threonine 937). (B) *In vitro* phosphorylation of wild-type Eg5 and Eg5_T937A_ with [γ^32^P]ATP in buffer in the presence (+) or absence (−) of Cdk1/cyclin B (top). Autoradiography (^32^P) and coomassie-stained polyacrylamide gel (C) are shown. Quantitative measurement of radioactivity of the corresponding bands from the SDS-gel. The degree of phosphorylation for wild-type Eg5 is 87.9% and is strongly reduced to 4.0% for Eg5_T937A_. (C) Phosphorylation does not affect the velocity of Eg5 as measured in microtubule gliding assays: Histograms of gliding velocities of individual microtubules (top) and bar plot of averaged gliding velocities (bottom) produced by wild-type Eg5 (WT) and a non-phosphorylatable mutant (Eg5_T937A_) treated with or without Cdk1 kinase. Red lines are a Gauss fit, error bars are standard errors. The velocities of phosphorylated and unphosphorylated wild-type Eg5 (WT) are not statistically significantly different (p = 0.067, significance level 0.05); the 12% difference between the velocities of wild-type Eg5 (WT) and the non-phosphorylatable mutant (T937A) is statistically significant (p = 8.6×10^−7^, significance level 0.05).

We generated an Eg5 mutant, in which threonine 937 in the known consensus site for Cdk1 phosphorylation was mutated to alanine (Eg5_T937A_, Fig. 1A). We performed radioactive phosphorylation experiments with purified Eg5 constructs *in vitro* and quantified the degree of phosphorylation using autoradiography and scintillation counting. Full-length wild-type and mutated Eg5 were incubated in buffer with [γ^32^P]ATP either in the absence or presence of Cdk1/cyclin B. Eg5_T937A_ was clearly less phosphorylated *in vitro* by Cdk1/cyclin B than wild-type Eg5 (Fig. 1B, top) confirming that threonine 937 is the major phosphorylation site of *Xenopus laevis* Eg5 for Cdk1 *in vitro*. Quantitative analysis (see Methods) revealed that the absolute degree of phosphorylation per Eg5 polypeptide chain was 87.9% for wild-type Eg5 in contrast to only 4.0% of residual phosphorylation for the mutant (Fig. 1B bottom).

Next we tested, if this phosphorylation affects the mechanical velocity of Eg5. We performed microtubule gliding assays, where surface adsorbed Eg5 propels fluorescently-labeled microtubules that are observed by time-lapse fluorescence microscopy. We found that the 1.7 µm/min velocity of phosphorylated Eg5 was not significantly different (T-test, p>0.05) from the 1.8 µm/min velocity of unphosphorylated Eg5 (Fig. 1C). The non-phosphorylatable mutant Eg5_T937A_ was also able to transport microtubules. The velocity of the mutant was 88% of that of wild-type Eg5, irrespective of the presence or absence of Cdk1/cyclin B (Fig. 1C). These results demonstrate that the non-phosphorylatable mutant of Eg5 is mechanochemically active and more importantly that phosphorylation of Eg5 by Cdk1/cyclin B does not affect the velocity of Eg5.

**Figure 2:**
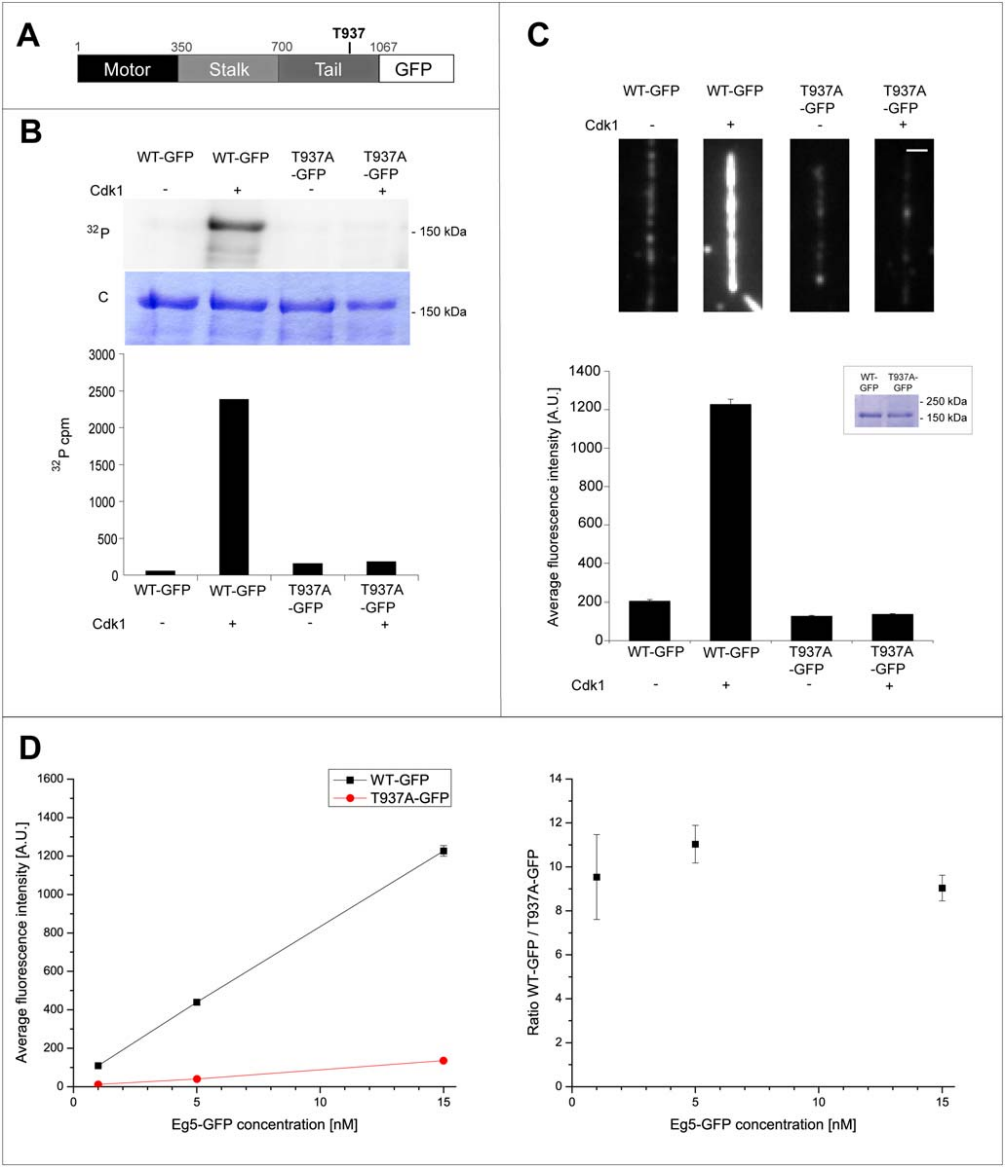
Phosphorylation of Eg5 by Cdk1 increases Eg5 binding to microtubules in buffer. (A) Schematic representation of the Eg5-GFP sequence. (B) *In vitro* phosphorylation of wild-type Eg5-GFP and Eg5_T937A_-GFP with [γ^32^P]ATP in buffer in the presence (+) or absence (−) of Cdk1/cyclin B. Autoradiography (^32^P) and coomassie-stained polyacrylamide gel (C) are shown. Quantitative measurement of radioactivity of the corresponding bands from the SDS-gel. The degree of phosphorylation of Eg5 wild-type is 75.6% and is strongly reduced to 5.9% for Eg5_T937A_-GFP. (C) Phosphorylation by Cdk1 increases the binding of Eg5 to microtubules *in vitro*: Representative examples for time-averaged images of the Eg5-GFP intensity on single immobilized microtubules as measured by TIRF microscopy (top). Wild-type Eg5-GFP (WT-GFP) and the non-phosphorylatable mutant (T937A-GFP) treated with ATP in the presence or absence of Cdk1/cyclin B were added to immobilized microtubules at a concentration of 15 nM. Scale bar is 1 µm. Bar plot of average Eg5-GFP signals (bottom) as obtained from 45–90 microtubules per condition as described above. Error bars are standard errors. Insert: Loading control showing the amount of Eg5-GFP in the phosphorylation reactions used in the experiment. (D) Average intensity signals (left) and intensity ratios (right) of Eg5-GFP and Eg5_T937A_-GFP on microtubules after Cdk1/cyclin B treatment plotted as a function of different Eg5 construct concentrations. For the average intensity signals (left), the values (in A.U.) are for WT and T937A at the concentration of 1 nM: 109±7 and 11±2, at the concentration of 5 nM: 439±17 and 40±3, at the concentration of 15 nM: 1226±39 and 136±8, respectively. Error bars are standard errors (in most cases obscured by the data symbol).

To test if phosphorylation affects the affinity of Eg5 for microtubules, we generated GFP fusions of wild-type and mutated Eg5 (Eg5-GFP and Eg5_T937A_-GFP, respectively, Fig. 2A top). Wild-type Eg5-GFP fusions were previously shown to be functional [30], [31]. We demonstrated that Eg5-GFP is specifically phosphorylated at T937 by Cdk1/cyclin B *in vitro* (Fig. 2B) as observed with Eg5 without GFP (Fig. 1B). Quantitative analysis (see Methods) demonstrated that after incubation with kinase, the degree of phosphorylation per Eg5 polypeptide chain was 75.6% for Eg5-GFP and only 5.9% for Eg5_T937A_-GFP (see Methods), corresponding to a 13-fold difference between wild-type and mutant (Fig. 2A bottom).

Next we measured the degree of binding of phosphorylated and unphosphorylated Eg5-GFP constructs to microtubules. We immobilized microtubules on functionalized glass coverslips, added GFP-tagged motors after phosphorylation reactions or control incubations and used total internal reflection fluorescence microscopy to quantify the steady state levels of Eg5-GFP constructs bound to individual microtubules (Fig. 2C top, see Methods). We found that in the presence of 15 nM motor, the amount of phosphorylated Eg5-GFP on microtubules was about 9-fold increased over the amount of non-phosphorylatable Eg5_T937A_-GFP incubated with kinase (Fig. 2C bottom). A similar result was obtained for the comparison between phosphorylated and unphosphorylated wild-type Eg5-GFP. The ratio in the amount of microtubule binding of wild-type to non-phosphorylatable mutant was constant in the range of the tested Eg5 concentrations between 1 and 15 nM (Fig. 2D). These results demonstrate that phosphorylation of threonine 937 by Cdk1 *in vitro* strongly enhances the binding of Eg5 to microtubules.

**Figure 3:**
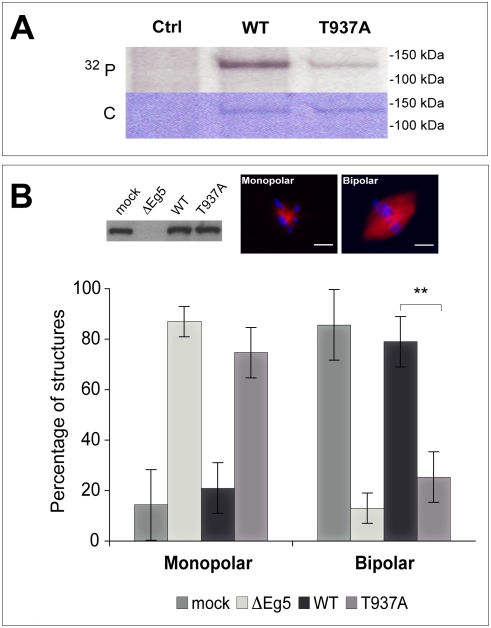
Spindles do not assemble in Eg5 depleted *Xenopus* egg extract in the presence of Eg5_T937A_. (A) Phosphorylation of wild-type Eg5 and of Eg5_T937A_ in mitotic *Xenopus* egg extract, showing a strong reduction of the degree of phosphorylation of Eg5_T937A_. As a control, an extract without recombinant Eg5 is shown (Ctrl). Autoradiography (^32^P) and Coomassie-stained polyacrylamide gel (C) are shown. (B) Western blot (top left) showing the amount of Eg5 in mock depleted extract (mock), in Eg5 depleted extract (ΔEg5) and in Eg5 depleted extract after addition of Eg5 wild-type (WT) or of Eg5_T937A_ (T937A). Fluorescence images of a monopolar spindle after Eg5 depletion (top middle) and of a bipolar spindle after ‘mock’ depletion (top right). Graphs (bottom) representing the percentages of monopolar and bipolar spindles under the conditions as indicated. More than 120 structures per condition were included in the analysis. ** indicates statistical difference (p = 0.0031, significance level 0.05). Spindle assembly cannot be rescued by Eg5_T937A_ at the concentration used. Microtubules are TAMRA-labeled (red) and DNA is stained with Hoechst (blue). Scale bar is 10 µm.

We then tested whether T937 in the Eg5 sequence is also phosphorylated by Cdk1 in mitotic *Xenopus* egg extract. Eg5_T937A_ and wild-type Eg5 were incubated in extract in the presence of [γ^32^P]ATP. Recombinant Eg5 constructs were immunoprecipitated and analyzed by autoradiography. The degree of phosphorylation of Eg5_T937A_ was reduced by about 90% as compared to wild-type Eg5 (Fig. 3A). These results demonstrate that Cdk1 is the major kinase that phosphorylates Eg5 in mitotic *Xenopus* egg extract.

We then investigated the role of phosphorylation of Eg5 by Cdk1 for bipolar spindle formation in *Xenopus* egg extract. We performed depletion/add back experiments and studied the effect of the replacement of endogenous Eg5 by mutated Eg5 on spindle assembly (Fig. 3B). Endogenous Eg5 was first immunodepleted from cytostatic factor (CSF) arrested egg extract. The extract was then supplemented with sperm nuclei and rhodamine-labeled tubulin, sent to interphase by addition of calcium and finally driven back to mitosis by addition of immunodepleted CSF extract containing recombinant Eg5 constructs. Efficient depletion of native Eg5 and replacement by recombinant Eg5 added at a concentration of about 400 nM, similar to the reported endogenous concentration [32], was confirmed by Western blot (Fig. 3B top left). Spindle structures were fixed after 45 min and analyzed by fluorescence microscopy. As expected, Eg5 depletion perturbed bipolar spindle formation, resulting in the formation of 80% monopolar spindles in contrast to the formation of 85% of bipolar spindles in control extracts that were ‘mock’ depleted with an irrelevant antibody (Methods) (Fig. 3B bottom). Replacement of native Eg5 by non-phosphorylatable Eg5_T937A_ resulted in the formation of 73% monopolar spindles very similar to the situation in Eg5 depleted extract (Fig. 3B bottom). Our results demonstrate that Cdk1 phosphorylation is essential for the function of Eg5 during spindle assembly in *Xenopus* egg extract.

Next we tested if a previously reported phosphorylation of *Xenopus* Eg5 somewhere in its stalk region by Aurora A is also essential for spindle assembly [22]. Using mass spectrometry of *in vitro* phosphorylated Eg5, we identified this site in *Xenopus* Eg5 and found it to be an evolutionary non-conserved serine at position 543 (Fig. S1, Text S1). We showed that phosphorylation at this site is not required for spindle formation in *Xenopus* egg extract (Fig. S1, Text S1). Therefore, Cdk1 is so far the only kinase known to be required for the mitotic function of Eg5.

Our observation that phosphorylation of Eg5 by Cdk1 strongly enhances its binding to microtubules in buffer (Fig. 2) suggests a simple explanation for previous reports regarding the targeting of Eg5 to spindles *in vivo*. It was shown that kinesin-5 mutated in the Cdk1 consensus site and overexpressed in HeLa cells [15] or in *Xenopus* A6 cells [7] does not localize to spindles in the presence of native kinesin-5. Similarly, only phosphorylated Eg5 was detected on spindles in *Drosophila* embryos using phospho-specific antibodies [17]. Monopolar spindles observed after Eg5 depletion and Eg5_T937A_ addition in *Xenopus* egg extract could therefore arise from a lack of Eg5 on microtubules.

**Figure 4:**
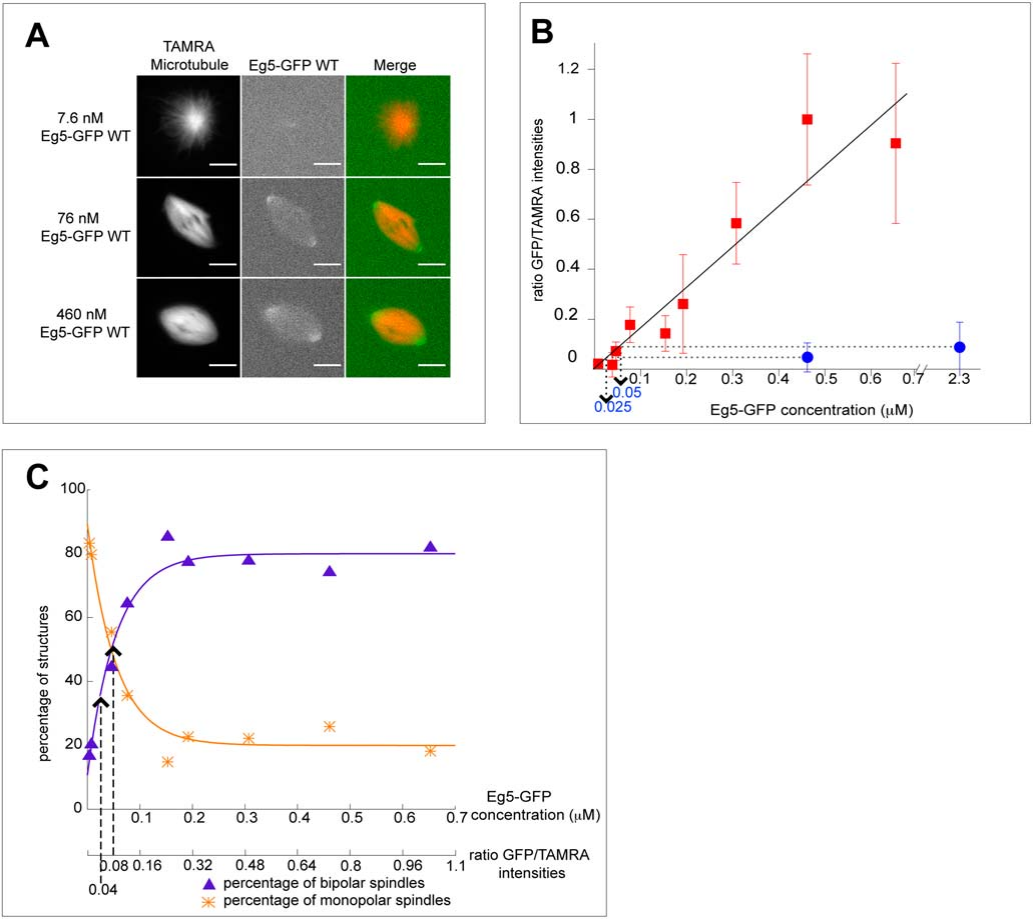
Cdk1 maintains the amount of Eg5 on spindle microtubules above a critical concentration required for spindle bipolarity. (A) Fluorescence images of different representative structures at varying Eg5-GFP concentrations added to Eg5 depleted extract. Fluorescence of TAMRA-labeled microtubules (left) and Eg5-GFP (middle) is shown together with merged images (right). Scale bar is 10 µm. (B) Graph representing the ratios of averaged GFP intensities per TAMRA intensities as a function of varying wild-type Eg5-GFP concentrations (red squares) and mutated Eg5_T937A_-GFP (blue circle). The ratios were normalized setting the ratio at 0.46 µM Eg5-GFP to 1. The line is a linear fit to the wild-type data (f(x) = 1.62 µM^−1^*x). Error bars indicate the standard error. (C) Graph illustrating the percentages of bipolar spindles (purple triangles) and of monopolar spindles (orange stars) as a function of the Eg5-GFP concentration. Depending on the condition, between 9 and 76 (on average 28) structures were counted per data point. The lines are fits to the data with using f(x) = 80%−69%*exp(−c/0.054 µM) for percentages of bipolar spindles and f(x) = 20%+69%*exp(−c/0.054 µM) for percentages of monopolar spindles. Concentrations on the x-axis were transformed into fluorescence ratios using the regression from B. The dashed lines indicate the fluorescence ratios for mutated Eg5_T937A_-GFP at the concentrations used in B.

We decided to quantify the amount of wild-type and mutated Eg5 on spindle microtubules in *Xenopus* egg extract. To be able to measure the amount of Eg5 using fluorescence, we replaced native Eg5 either by recombinant Eg5-GFP or Eg5_T937A_-GFP. This ensured also that native Eg5 does not compete with Eg5_T937A_ for microtubule binding, in contrast to previous experiments [15]. The biological functionality of Eg5-GFP was confirmed by its ability to rescue spindle formation in Eg5 depleted *Xenopus* egg extract (Fig. 4A), as shown previously [30], [31]. As expected from our results above, addition of Eg5_T937A_-GFP to depleted extract failed to rescue bipolar spindle formation. Moreover, the construct with the deficient Cdk1 phosphorylation site was only extremely weakly bound to the microtubules of monopolar spindles. Quantification based on fluorescence intensities of the GFP constructs of Eg5 and of the rhodamine-labeled microtubules demonstrated that the amount of the mutant bound to microtubules is about 20 times lower than that of wild-type Eg5, if added to extract at endogenous concentrations (Fig. 4B).

To test if this reduction of Eg5 on spindle microtubules is sufficient to explain the failure of spindle formation, we titrated the amount of wild-type Eg5-GFP in depleted extract and measured both the percentage of spindle recovery and the amount of Eg5-GFP in spindle structures, a quantitative experiment for which the *Xenopus* egg extract system is well suited. We found that the percentage of bipolar spindles increased to a maximum value of about 80%, when a critical concentration of about 0.2 µM Eg5-GFP in the extract was reached. Above this concentration, the efficiency of spindle formation remained constant (Fig 4C). Interestingly, this was not reflected by the amount of Eg5 in the spindle that continued to increase when the concentration of Eg5 in the extract was increased above the critical concentration (Fig. 4B). This shows that Eg5 does not saturate the available binding sites on spindle microtubules at the critical concentration. The increase of Eg5 on spindle microtubules beyond the critical amount does not disturb spindle formation, which might indicate that the Eg5 molecules do not experience a strong antagonistic force above the critical concentration. This agrees with the observation that the velocity of microtubule flux in the spindle is similar to Eg5 driven anti-parallel sliding of microtubules *in vitro* [18], [19]. It is furthermore interesting to note that the native concentration of Eg5 in extract is above this critical concentration required for bipolarity. This might ensure robust spindle formation. The existence of a critical concentration for bipolarity might be a conserved feature of spindles in higher eukaryotes, as the efficiency of bipolar spindle formation was also found to be dependent on the amount of kinesin-5 on the spindle in *Drosophila* S2 cells [20].

When native Eg5 was replaced by non-phosphorylatable Eg5_T937A_-GFP at roughly the endogenous concentration, the amount localizing to the spindles was clearly below the critical amount required for spindle formation (Fig. 4B, C). At this concentration mostly monopolar spindles formed (Fig. 3C). Increasing the concentration of Eg5_T937A_-GFP to 4–5 times the endogenous concentration increased the amount of the mutant bringing it now closer to the critical amount leading to an increase in bipolar spindle formation. This result illustrates that the phosphorylation of Eg5 by Cdk1 controls also the apparent affinity of Eg5 for spindle microtubules in *Xenopus* egg extract, thereby ensuring that the amount of Eg5 in the spindle when present at the endogenous concentration in the extract is well above the critical value required for spindle bipolarity.

## Discussion

Eg5 is a member of the kinesin-5 subfamily of kinesin-like proteins having a conserved phosphorylation site for Cdk1 in the tail of the molecule that is typical for this subfamily [14]. Kinesin-5 localizing to spindles in *Drosophila* embryos was reported to be phosphorylated at this conserved site [17]. Mutated kinesin-5 molecules without this conserved phosphorylation site do not localize to spindles when overexpressed in vertebrate cells [7], [15]. It was furthermore shown that expression of a non-phosphorylatable kinesin-5 mutant in *Drosophila* S2 cells after a native kinesin-5 knock-down did not bind to microtubules in mitosis and consequently led to monoastral spindles [20]. This finding was in contrast to a result in *Schizosaccharomyces pombe*, where phosphorylation of the Eg5 ortholog was found not to be essential for spindle function [21].

We addressed here the question of Eg5 targeting to microtubules by Cdk1 quantitatively both in buffer and in *Xenopus* egg extract. We found that the efficiency of binding of purified full-length Eg5 to microtubules in buffer increases strongly upon phosphorylation at threonine 937. Hence, this increase in binding efficiency is an intrinsic property of Eg5. Apart from a strongly reduced propensity to bind microtubules, non-phosphorylated wild-type Eg5 as well as a non-phosphorylatable Cdk1 mutant of Eg5 are mechanochemically functional as judged by their microtubule gliding activity *in vitro*. The minor, but significant decrease of the speed of the mutant as observed in our gliding assays is not expected to cause a drastic effect on spindle formation, because Eg5 mutants with reduced speeds were shown in the past to lead to the assembly of bipolar spindles in *Xenopus* egg extract [33].

We compared our results with purified Eg5 in buffer with its behavior in the presence of all its potential binding partners, using spindle reconstitutions in *Xenopus* egg extract. We first established that there is a critical concentration of Eg5 in *Xenopus* egg extract that is required for the formation of spindles, similar to an earlier observation in *Drosophila* S2 cells [34]. Using *Xenopus* egg extract allowed us to determine the absolute value of this concentration which we found to be 0.2 µM. This threshold concentration is well below the endogenous concentration of Eg5 in *Xenopus* egg extract [32] ensuring robust spindle formation under native conditions. After depletion of endogenous Eg5 from egg extract and addition of mutated Eg5 deficient for phosphorylation by Cdk1, we found that this mutant localized to microtubules in amounts that are far below the critical concentration that is required for bipolar spindle formation, unless added at unnaturally elevated concentrations. This explains why this mutant does not support spindle assembly.

Cdk1 activity therefore regulates the amount of Eg5 binding to spindle microtubules throughout mitosis in all metazoans tested so far for Eg5 function. In metaphase, when the kinase activity is high, Cdk1 ensures that the amount of Eg5 on microtubules is above the critical level required for bipolar spindle formation. This explains also why Eg5 was reported to localize only in low amounts to spindle microtubules in anaphase, when Cdk1 activity decreases [15]. This behavior appears to be conserved only in higher eukaryotes. The situation was shown to be different in fission yeast, where phosphorylation of kinesin-5 by Cdk1 is non-essential [21].

The positive regulation of Eg5 binding to microtubules by Cdk1 dependent phosphorylation is remarkable. In most cases phosphorylations of motors [8], [20], [35] or of non-motile microtubule crosslinking proteins [36] by Cdk1 negatively regulate binding to microtubules *in vivo*. It has been unclear if the positive regulation of microtubule binding by Cdk1 phosphorylation in the case of Eg5 is a consequence of an interaction of Eg5 with other binding partners also localizing to microtubules such as the dynactin component p150^Glued^ to which Eg5 was reported to bind in a Cdk1 phosphorylation dependent manner [37]. Our results show that the stimulation of Eg5 binding to spindle microtubules is, at least in part, a direct consequence of an intrinsic increase of the ability of Eg5 to bind to microtubules upon phosphorylation at the conserved Cdk1 phosphorylation site in its tail region. The local increase of the amount of Eg5 on spindle microtubules upon phosphorylation is expected to also contribute to promote the interaction of Eg5 with other components localizing to the spindle such as p150^Glued^ of the dynactin complex [31], [37].

In their combination, several previous reports [22]–[25], [28], [38] gave rise to the hypothesis that the activity of the motor protein Eg5 could also be locally regulated by the small GTPase Ran through a pathway involving TPX2-stimulated phosphorylation in the stalk of Eg5 by Aurora A. We identified a site that is phosphorylated *in vitro* by Aurora A in the stalk of Eg5 as previously reported [22]. We found that its phosphorylation is not required for spindle formation in *Xenopus* egg extract (Fig. S1, Text S1), which is in agreement with this site not being evolutionary conserved.

In conclusion, the only direct phosphorylation currently having been experimentally verified to be important for the function of Eg5 during spindle assembly is phosphorylation by Cdk1 of a conserved threonine in the C-terminal tail of Eg5. The presence of a phosphate at this position positively regulates directly the binding of Eg5 to microtubules. This ensures efficient binding of this motor to microtubules selectively during mitosis when the activity of Cdk1 is high, hence ensuring spindle formation.

## Materials and Methods

### Cloning

The complete sequence of *Xenopus laevis* Eg5 (corresponding to the complete reading frame from amino acid 1 to 1067, gift of Claire Walczak) was amplified by PCR and inserted into pFASTBacHTa (Invitrogen) using the SalI and NotI sites yielding a construct with a N-terminal hexahistidine tag. A threonine 937 to alanine substitution was introduced using Quickchange 2 XL (Stratagene) generating Eg5_T937A_. Constructs of wild-type Eg5 and Eg5_T937A_ carrying an additional GFP at their C-terminus (Eg5-GFP and Eg5_T937A_-GFP) were generated as described elsewhere [31].

### Protein expression and purification

The Eg5 constructs were expressed in Sf9 insect cells and purified as follows. Cells were lysed in lysis buffer (50 mM KH_2_PO_4_, 250 mM KCl, 10 mM imidazole, 0.5 mM MgATP, 0.1% Triton ×100, 10 mM β-mercaptoethanol, protease inhibitors (Roche), pH 8.0) and centrifuged at 176,000 g for 30 min at 4°C. The clarified supernatant was incubated with Talon resin (Clontech) for 1 h at 4°C. The resin was washed with washing buffer (50 mM KH_2_PO_4_, 250 mM KCl, 10 mM imidazole, 0.1 mM MgATP, 10% glycerol, protease inhibitors (Roche), 10 mM β-mercaptoethanol, pH 8.0) and protein was eluted with elution buffer (50 mM KH_2_PO_4_, 150 mM KCl, 250 mM imidazole, 0.1 mM MgATP, 10% glycerol, protease inhibitors (Roche), pH 7.0). The protein was dialyzed overnight in dialysis buffer (50 mM KCl, 50 mM imidazole, 0.5 mM EGTA, 10% sucrose, 10 mM β-mercaptoethanol, pH 7.0) at 4°C. For proteins used for add back experiments, the β-mercaptoethanol was omitted from the dialysis buffer, which was instead degassed, and the dialysis lasted only for 3 h. Finally, aliquots were frozen in liquid ethane and stored in liquid nitrogen. For the *in vitro* binding experiments, the GFP fusion proteins were furthermore purified using size-exclusion chromatography with a Superose-6 column on an FPLC (GE Biosciences).

Polyclonal anti-Eg5 antibody was purified from the serum of a rabbit (Eurogentec) injected with GST-Eg5 (constructed and purified as described [13]) using an antigen affinity column (Eg5 coupled to NHS-activated HiTrap (Amersham)). For loading, the serum was recirculated over the column overnight at 4°C. The column was then washed with washing buffer (500 mM NaCl, 0.1% Triton ×100, phosphate-buffered saline (PBS)) followed by elution with 100 mM glycine pH 2.6 into Eppendorf tubes containing 10% (v/v) of 2 M Tris pH 8.0. The antibody was dialyzed overnight into PBS/50% glycerol and stored at −20°C.

### Phosphorylation experiments in buffer

*In vitro* phosphorylation of full length Eg5 either with or without a carboxy-terminal GFP tag were performed by incubating 300 nM of Eg5 construct in 20 µl kinase assay buffer (100 mM KCl, 50 mM imidazole, 3 mM MgCl_2_, 50 mM HEPES, 2 mM DTT, 200 mM sucrose, 0.5 mM EGTA, pH 7) containing 10 mM MgATP, 3 µM β-casein (Sigma), 20 nM Cdk1/cyclinB (Cell Signaling), phosphatase inhibitors (100 mM NaF, 80 mM glycerophosphate, 1 mM microcysteine), and for radioactive phosphorylation experiments additionally 0.5 mCi/ml [γ^32^P]ATP (Amersham). The sample was incubated for 30 min to 1 h at room temperature. The phosphorylation reactions were boiled in SDS gel sample buffer and loaded on a 10% SDS-polyacrylamide gel. The gel was stained with Coomassie Brillant Blue, dried and exposed to a phosphorimager FLA7000 film (Fujifilm). Bands corresponding to Eg5 were excised from the gel and the amount of radioactivity was quantified using a scintillation counter (Beckmann). The percentage of phosphorylated Eg5 was calculated based on the known amount of Eg5 per band and the measured radioactivity taking into account the specific activity of [γ^32^P]ATP at the time of measurement.

### Microtubule gliding assay

Alexa-568 (Invitrogen) labeled microtubules were generated by polymerizing 10 mg/ml purified tubulin [39] with 2 mg/ml Alexa-568-labeled tubulin and 1 mM GTP in BRB80 (80 mM PIPES pH 6.8, 1 mM MgCl_2_, 1 mM EGTA). After a tenfold dilution in BRB80 containing 20 µM paclitaxel (Sigma), microtubules were pelleted for 5 min in a tabletop centrifuge at 14,000 rpm (Eppendorf 5417C). The microtubule pellet was resuspended in BRB80/paclitaxel.

A flow chamber with a volume of about 5 µl was built by assembling two glass coverslips on top of each other using double sticky tape (Tesa). The flow chamber was first washed with assay buffer (72 mM Pipes, 0.9 mM MgCl2, 0.9 mM EGTA, 50 mM KCl, 20 µM paclitaxel, 14 mM 2-mercaptoethanol, 1% Glucose, 2 mM Mg-ATP), followed by 300 nM motor in kinase assay buffer (see above). The motor was incubated 10 min on ice and 2 min at room temperature, and then the flow chamber was washed with 4 flow chamber volumes of assay buffer. Finally, motility buffer (assay buffer supplemented with 0.1 mg/ml β-casein, 0.4 mg/ml glucose oxidase (Serva), 0.2 mg/ml catalase (Sigma) and microtubules diluted 1∶2000) was flowed in.

For imaging, an inverted microscope (as described above) was used. Usually sequences of 2.5 min time lapse were recorded with 1 frame taken every 5 s and 500 ms exposure time per frame. The experiment was performed at 21°C. 4 time-lapse movies per condition were analyzed with the Kymograph plugin of ImageJ [40]. For statistical analysis a t-test was performed.

### Quantification of binding of Eg5-GFP to individual microtubules *in vitro*

Alexa568- and biotin-labeled microtubules were polymerized in presence of GMPCPP (Jena Bioscience) as described [41]. Microtubules were attached in a 5 µl flow chamber to a PEG-biotin coated glass slide via neutravidin as described [42]. Motor treated with or without kinase was diluted to the given concentrations in a buffer containing 40 mM PIPES pH 6.8, 0.5 mM EGTA, 0.5 mM MgCl_2_, 10 µM paclitaxel, 10 mM 2-mercaptoethanol, 1% glucose, 1 mg/ml glucose oxidase (Serva), 0.5 mg/ml catalase (Sigma) and 1 mM Mg-ATP and flowed into the chamber. Total internal reflection fluorescence (TIRF) microscopy was performed on an Olympus TIRF microscope [42] at 160× magnification. Camera and laser settings as well as the temperature (20°C) were kept constant during the entire set of experiments. Streams of 500 frames at 100 ms exposure time were recorded in the GFP channel. The position of the microtubules was recorded in the Alexa568 channel.

Eg5-GFP intensities were calculated based on average Z-projections of these 500 frames. The intensity of the Eg5-GFP signal on a microtubule was determined as the difference between the signal along a microtubule and the background signal along a line directly next to it (ImageJ, Rasband, W.S, NIH). The averaged intensity signals were obtained from 45–90 of such background-subtracted individual intensity signals per condition.

### Phosphorylation experiments in *Xenopus* egg extract

1 µg of recombinant Eg5 construct was incubated in 20 µl *Xenopus* egg extract containing 15 mCi/ml radioactive [γ^32^P]ATP (Amersham) for 30 min at room temperature. The phosphorylation reaction was diluted with 20 µl CSF-XB supplemented with phosphatase inhibitors (100 mM NaF, 80 mM glycerophosphate, 1 mM PMSF, 20 mM EDTA, 1 mM sodium vanadate, 1 mM microcysteine) and protease inhibitors (Roche) and then incubated for 2 h on ice with anti-pentahistidine antibody (Qiagen) saturated protein G Dynabeads. After retrieval of the Dynabeads with immunoprecipitated Eg5 using a magnet, the beads were boiled in SDS gel sample buffer and loaded on a 10% SDS-polyacrylamide gel. The gel was exposed to a Kodak Biomax film. The phoshorylation signals were quantified using Image J. First, the respective signal intensities on the autoradiograph and on the Coommasie stained SDS-PAGE gel were measured. After background subtraction, the ratio of these two intensities was calculated for wild-type and mutant Eg5. Two independent experiments were performed in different extracts and were averaged.

### Spindle assembly in *Xenopus* egg extract

Cytostatic factor arrested extract (CSF extract) was prepared from *Xenopus laevis* eggs as described previously [43] except that eggs were packed for 1 min at 600 g, then for 1 min at 1200 g and finally crushed at 10,000 g for 18 min at 16°C (slow acceleration). Spindle assembly was performed as described [44]. Briefly, tubulin labeled with tetramethylrhodamine (TAMRA) (Molecular Probes) [45] and purified, demembranated sperm nuclei [46] were added to CSF extract, half of which was sent to interphase with calcium solution (0.4 mM CaCl_2_, 10 mM KCl, 0.1 mM MgCl_2_), while the other half was kept on ice. After usually 60 min, interphasic nuclei were observed and the extract was cycled back to mitosis with the addition of the other half of CSF extract.

For depletion/add back experiments, 80 µl of protein A Dynabeads coupled to anti-Eg5 antibody, or in controls an irrelevant IgG (‘mock’ depletion), were used to deplete 100 µl extract for 1 h on ice. Spindle assembly was performed as described above. Recombinant proteins or buffer as a control were added with depleted CSF extract as the reaction was cycled back to mitosis. The depletion efficiency and the concentration of Eg5 after add back were determined by Western Blot from 0.5 µl extract loaded onto a SDS gel. Extracts were diluted after 45 min with dilution buffer (30% glycerol, 0.5% Triton ×100 in BRB80 (80 mM PIPES, 1 mM MgCl_2,_ 1 mM EGTA, pH 6.8)), fixed with fixation buffer (dilution buffer with 4% formaldehyde) and layered on top of a 3 ml cushion (40% glycerol in BRB80). After centrifugation at 3000 rpm for 30 min at 20°C (Heraeus centrifuge), coverslips were post-fixed in methanol at −20°C. Then they were rehydrated, washed in PBS/0.1% Triton ×100, and finally mounted. In some experiments Hoechst (H33342, Sigma) was used to stain DNA prior mounting (Fig. 3A). Images were taken using an inverted microscope (Axiovert 135TV, Zeiss, 100 W mercury lamp, CDD camera Coolsnap HQ (Photometrics)) with a 63× oil immersion objective (Zeiss).

For the quantification of the percentages of monopolar and bipolar spindles, randomly selected structures were counted from at least 3 different experiments and the results per condition were averaged. For statistical analysis, the equality of the variances was first tested with a Levene's test for unparametric and unpaired data. As the variances were found equal, a t-test was performed thereafter.

### Quantification of Eg5 in spindles

To quantify the amount of Eg5 on spindle microtubules, fluorescence images of fixed spindles formed in the presence of Eg5-GFP fusions were taken as described above. Using ImageJ, the mean intensity per area was measured in a region of interest drawn around randomly selected structures. After subtraction of background measured in areas not containing microtubules, the GFP and TAMRA intensities of at least 10 structures were averaged (except for the addition of 46 nM Eg5-GFP for which 6 structures were analyzed). From the averaged GFP intensities an averaged background signal of spindles in mock depleted extract not containing Eg5-GFP was then subtracted. Typically, 2 or 3 different conditions corresponding to different concentrations of added Eg5-GFP were analyzed per extract, and in total 6 different extracts were used. As the measured TAMRA fluorescence intensity of spindles varied from extract to extract, we used the total average of TAMRA intensities from spindle structures in all extracts divided by the average TAMRA intensity of spindle structures per extract to correct the measured TAMRA values of each spindle in order to be able to compare experiments performed in different extracts. Finally, the ratios of GFP intensities to corrected TAMRA intensities were calculated and then normalized so that the ratio was set to 1 at the Eg5-GFP concentration corresponding to 460 nM, which is roughly the endogenous concentration [32]. Fits to the data of Figs. 4B and C were performed using gnuplot.

## Supporting Information

Text S1 Results and Discussion S1, Methods S1, References S1
(0.12 MB PDF)

Figure S1 Aurora A phosphorylation on serine 543 of Eg5 is not required for spindle assembly in Xenopus egg extract. (A) Schematic representation of the GST-Eg5ΔC sequence with the phosphorylation site for Aurora A (S543) in the stalk (left). Phosphorylation of wild-type GST-Eg5ΔC (WT) and GST-Eg5S543AΔC by Xenopus Aurora A (Aur A) with [γ32P]ATP in buffer (right). Coomassie-stained polyacrylamide gel (C) and autoradiography (32P) are shown. (B) Schematic representation of the full-length Eg5 sequence with the phosphorylation sites for Aurora A (S543) in the stalk (top). Phosphorylation of wild-type Eg5′ (WT′) and Eg5′S543A in the presence (+) or absence (−) of Aurora A (Aur A) in buffer (left) (the symbol ′ indicates a modification in the N-terminal tag of the Eg5 construct, see Methods S1). Phosphorylation of wild-type Eg5′ and mutated Eg5′S543A in mitotic Xenopus egg extract (right). As a control, an extract without recombinant Eg5 is shown (Ctrl). Coomassie-stained polyacrylamide gels (C) and autoradiographies (32P) are shown. (C) Western blot (insert) showing the amount of Eg5 in mock depleted extract (mock), in Eg5 depleted extract (ΔEg5) and in Eg5 depleted extract after addition of Eg5′ wild-type (WT′) or of Eg5′S543A (S543A′). Graphs representing the percentages of monopolar and bipolar spindles formed in extract. * indicates no statistical difference (p = 0.97, significance level 0.05).
(2.03 MB PDF)
