# Supplementary material for: Phosphorylation by Cdk1 Increases the Binding of Eg5 to Microtubules In Vitro and in Xenopus Egg Extract Spindles
Source: PLoS One. 2008 Dec 15;3(12):e3936. doi: 10.1371/journal.pone.0003936 (PMC2592692; doi:10.1371/journal.pone.0003936)
Supplement: Text S1 — Results and Discussion S1, Methods S1, References S1 (0.12 MB PDF) [file pone.0003936.s001.pdf]

TEXT S1

RESULTS AND DISCUSSION S1

Previously *Xenopus laevis* Eg5 was reported to be phosphorylated by Aurora A *in vitro* somewhere in the central part (the stalk) of its sequence [1]. We decided to investigate the role of this phosphorylation. Using mass spectrometry analysis, we identified serine 543 as the site in the stalk of Eg5 that is phosphorylated by Aurora A *in vitro* (Methods S1). The coverage of the Eg5 sequence by this analysis was above 80% and no other site was identified to be phosphorylated in the covered region. We generated single point mutants with a serine 543 to alanine substitution. A truncated construct lacking the C-terminal 413 amino acids of Eg5 (GST-$Eg5_{S543A}\Delta C$), being similar to the construct used in the previous Aurora A phosphorylation study [1] was 99-fold less phosphorylated by Aurora A *in vitro* than the truncated wild-type protein (GST-Eg5ΔC), as demonstrated by autoradiography of immunoprecipitated Eg5 phosphorylated in the presence of radioactive [γ^{32}P]ATP and Aurora A (Fig. S1A). This demonstrates that serine 543 is the major phosphorylation site of truncated Eg5 from *Xenopus laevis* for Aurora A *in vitro*. Sequence comparison revealed, however, that this site is not conserved among other kinesin-5 family members.

A full-length mutant ($Eg5_{S543A}$) was also less phosphorylated by Aurora A in buffer than full-length wild-type Eg5, however only by a factor of 2.2 (Fig. S1B left), leaving the possibility that there is an additional site in full-length Eg5 for *in vitro* phosphorylation by Aurora A that was not identified in our mass spectrometry analysis despite the 80% coverage. In *Xenopus* egg extract, both S543A mutated and wild-type full-length Eg5 were similarly phosphorylated (differing by a factor of only 1.3) (Fig. S1B right), indicating that serine 543 is not a major phosphorylation site of Eg5 in *Xenopus* egg extract.

Depletion of native Eg5 from *Xenopus* egg extract and replacement by either recombinant wild-type Eg5 or $Eg5_{S543A}$ lead in both cases to spindle formation (Fig. S1C). No evident changes in Eg5 localization or spindle morphology were observed in the presence of $Eg5_{S543A}$. This demonstrates that phosphorylation of Eg5 at serine 543 by Aurora A is not essential for the function of Eg5 during spindle assembly in *Xenopus* egg extract. Therefore, the proposed regulatory effect of Ran.GTP on the activity of Eg5 in *Xenopus* egg extract [2] is not mediated through phosphorylation by Aurora A at serine 543 of Eg5. However, it is still possible that Aurora A regulates Eg5 via an alternative mechanism.

METHODS S1

Mass spectrometry analysis

In vitro phosphorylation reactions for mass spectrometry analysis were performed by incubating 0.2 mg/ml full-length Eg5 with 0.14 mg/ml Aurora A and 10 mM MgATP for 20 min at 37°C in phosphorylation buffer (a 1:1 mixture of Eg5 dialysis buffer (Materials and Methods) and kinase buffer (50 mM Tris pH 7.5, 50 mM NaCl, 1 mM DTT, 12 mM $MgCl_2$). As negative control the same reaction was performed in the absence of kinase. Mixtures containing 4 µg Eg5 were separated on a SDS polyacrylamide gel. Gel-excised Eg5 samples were digested with trypsin. The phosphorylated peptides were enriched using home-made TiO_2 tips [3]. The digested samples were analyzed by LC/ ESI MS in the precursor ion discovery (PID) mode [4] with and without TiO_2 enrichment. Each sample contained 100 mM EDTA [5]. Mass spectrometry analysis was performed on a Q-TOF Global (Micromass) equipped with a Z-spray source and operating in positive ion mode. Data were acquired using MassLynx 4.0. Calibration was performed using an MS/MS spectrum of Glu-fibrinopeptide B. Tryptic peptides were separated on-line using nano liquid chromatography (Precolumn: 5 x 0.5 mm C18, LC Packings, analytical column: 75 µm x 150 mm, waters) on a Waters CapLC system using a linear gradient with H_2O (solvent A), acetonitrile (solvent B), with increasing solvent B from 5% to 40% over 90 min. MS data were acquired the range of 400-1800 m/z in the MS mode and 50-1800 m/z in the MS/MS mode. For PID experiments, the collision energy (CE) was switched between low (10 V) and high (31 V) every second. MS/MS data acquisition was triggered by the neutral loss 97.9799. A single peptide has a confirmed phosphorylation site. This peptide corresponds to residues 541-555 (sequence RF**pS**VIQQTVDEYSVK) with phosphorylation at serine 543. We also observed an additional phosphorylation site at the N-terminal (RM**pS**SQNSFMSSK) with phosphorylation at serine 2 in the various forms of this peptide resulting from miscleavages and levels of methionine oxidation. Replacement of the two arginines in the pFasctBac-encoded tag that are closest to the N-terminal of the Eg5 sequence removed the artificial consensus sequence generated by the tag and phosphorylation at this site was no longer detected (data not shown). The coverage of the Eg5 sequence in the mass spectrometry analysis was above 80%. Phosphorylation at serine 543 was detected in three independently prepared samples of Eg5 phosphorylated by Aurora A.

Cloning, expression and protein purification

A single point serine 543 to alanine substitution was introduced into full-length Eg5 in pFASTBacHTa and into truncated GST-Eg5ΔC (with amino acids 1 - 654 of the Eg5 sequence) in pGEX4T3 using Quickchange 2 XL (Stratagene) yielding the two constructs $Eg5_{S543A}$ and GST-$Eg5_{S43A}$ΔC. Two additional arginine to glycine substitutions were made in the N-terminal tag of the pFASTBacHTa vector at positions -1 and -2 before the start of the Eg5 sequence (Eg5' and $Eg5'_{S543A}$ in Fig. S1). This prevented the generation of an artificial Aurora A phosphorylation site in the beginning of the Eg5 sequence (as demonstrated by mass spectrometry analysis, data not shown). Full-length Eg5 constructs were expressed in Sf9 insect cells and purified as described in Materials and Methods. Truncated GST-Eg5ΔC constructs were expressed in *E. coli* and purified as described [6].

Autoradiography of Eg5 phosphorylated by Aurora A in buffer and in *Xenopus* egg extract

In vitro phosphorylation reactions with Aurora A were performed by incubating 1 μg of full-length Eg5', $Eg5'_{S543A}$, truncated GST-Eg5ΔC or GST-$Eg5_{S43A}$ΔC in 20 μl CSF-XB (100 mM KCl, 0.1 mM $CaCl_2$, 3 mM $MgCl_2$, 10 mM HEPES, 50 mM sucrose, 5 mM EGTA, pH 7.7) containing 10 mM MgATP, 0.75 mCi/ml radioactive [γ^{32}P]ATP (Amersham) and 0.2 μg of Aurora A for 30 min at room temperature. The phosphorylation reaction was diluted with 20 μl CSF-XB supplemented with phosphatase inhibitors (100 mM NaF, 80 mM glycerophosphate, 1 mM PMSF, 20 mM EDTA, 1 mM sodium vanadate, 1 mM microcysteine) and protease inhibitors (Roche) and then incubated for 2 h on ice with antibody-saturated protein G Dynabeads. Anti-GST antibodies (Molecular Probes) were used for GST-tagged truncated Eg5 constructs, anti-pentahistidine antibody (Qiagen) for histidine-tagged full length Eg5 constructs. After retrieval of the Dynabeads with immunoprecipitated Eg5 using a magnet, the beads were boiled in SDS gel sample buffer and loaded on a 10% SDS-polyacrylamide gel. The gel was exposed to a Kodak Biomax film. The same protocol was followed for phosphorylation experiments in mitotic egg extract, except that phosphorylation reactions were performed in *Xenopus* egg extract (see below) with a final concentration of 15 mCi/ml [γ^{32}P]ATP.

The phoshorylation signals were quantified using Image J. First, the respective signal intensities on the autoradiograph and on the Coommasie stained SDS-PAGE gel were

measured. After background subtraction, the ratio of these two intensities was calculated for wild-type and mutant Eg5.

REFERENCES S1

1. Giet R, Uzbekov R, Cubizolles F, Le Guellec K, Prigent C (1999) The Xenopus laevis aurora-related protein kinase pEg2 associates with and phosphorylates the kinesin-related protein XlEg5. J Biol Chem 274: 15005-15013.
2. Wilde A, Lizarraga SB, Zhang L, Wiese C, Gliksman NR, et al. (2001) Ran stimulates spindle assembly by altering microtubule dynamics and the balance of motor activities. Nat Cell Biol 3: 221-227.
3. Larsen MR, Thingholm TE, Jensen ON, Roepstorff P, Jorgensen TJ (2005) Highly selective enrichment of phosphorylated peptides from peptide mixtures using titanium dioxide microcolumns. Mol Cell Proteomics 4: 873-886.
4. Bateman RH, Carruthers R, Hoyes JB, Jones C, Langridge JI, et al. (2002) A novel precursor ion discovery method on a hybrid quadrupole orthogonal acceleration time-of-flight (Q-TOF) mass spectrometer for studying protein phosphorylation. J Am Soc Mass Spectrom 13: 792-803.
5. Liu S, Zhang C, Campbell JL, Zhang H, Yeung KK, et al. (2005) Formation of phosphopeptide-metal ion complexes in liquid chromatography/electrospray mass spectrometry and their influence on phosphopeptide detection. Rapid Commun Mass Spectrom 19: 2747-2756.
6. Sawin KE, LeGuellec K, Philippe M, Mitchison TJ (1992) Mitotic spindle organization by a plus-end-directed microtubule motor. Nature 359: 540-543.
