# Supplementary figures and images for: Phosphorylation by Cdk1 Increases the Binding of Eg5 to Microtubules In Vitro and in Xenopus Egg Extract Spindles
Source: PLoS One. 2008 Dec 15;3(12):e3936. doi: 10.1371/journal.pone.0003936 (PMC2592692; doi:10.1371/journal.pone.0003936)

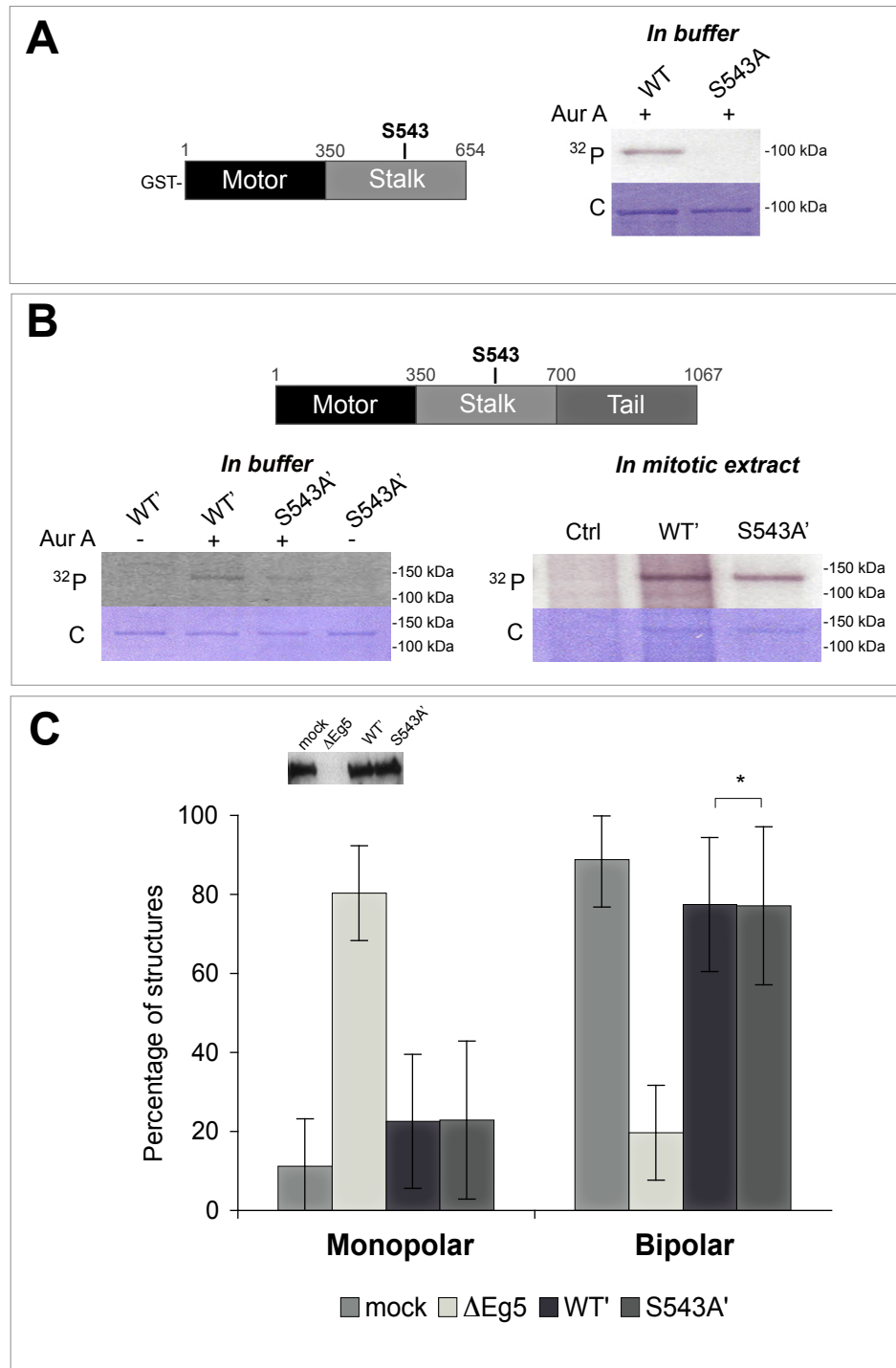

**Figure S1**

Supplement: Figure S1 — Aurora A phosphorylation on serine 543 of Eg5 is not required for spindle assembly in Xenopus egg extract. (A) Schematic representation of the GST-Eg5ΔC sequence with the phosphorylation site for Aurora A (S543) in the stalk (left). Phosphorylation of wild-type GST-Eg5ΔC (WT) and GST-Eg5S543AΔC by Xenopus Aurora A (Aur A) with [γ32P]ATP in buffer (right). Coomassie-stained polyacrylamide gel (C) and autoradiography (32P) are shown. (B) Schematic representation of the full-length Eg5 sequence with the phosphorylation sites for Aurora A (S543) in the stalk (top). Phosphorylation of wild-type Eg5′ (WT′) and Eg5′S543A in the presence (+) or absence (−) of Aurora A (Aur A) in buffer (left) (the symbol ′ indicates a modification in the N-terminal tag of the Eg5 construct, see Methods S1). Phosphorylation of wild-type Eg5′ and mutated Eg5′S543A in mitotic Xenopus egg extract (right). As a control, an extract without recombinant Eg5 is shown (Ctrl). Coomassie-stained polyacrylamide gels (C) and autoradiographies (32P) are shown. (C) Western blot (insert) showing the amount of Eg5 in mock depleted extract (mock), in Eg5 depleted extract (ΔEg5) and in Eg5 depleted extract after addition of Eg5′ wild-type (WT′) or of Eg5′S543A (S543A′). Graphs representing the percentages of monopolar and bipolar spindles formed in extract. * indicates no statistical difference (p = 0.97, significance level 0.05). (2.03 MB PDF) [file pone.0003936.s002.pdf]
